# A cocktail of p16^INK4a^ and Ki-67, p16^INK4a^ and minichromosome maintenance protein 2 as triage tests for human papillomavirus primary cervical cancer screening

**DOI:** 10.18632/oncotarget.19870

**Published:** 2017-08-03

**Authors:** Hai-Rui Wang, Yu-Cong Li, Hui-Qin Guo, Lu-Lu Yu, Zeni Wu, Jian Yin, Guang-Dong Liao, Yi-Min Qu, Yu Jiang, Dong Wang, Wen Chen

**Affiliations:** ^1^ Department of Cancer Epidemiology, National Cancer Center, Cancer Hospital, Chinese Academy of Medical Sciences and Peking Union Medical College, Beijing, PR China; ^2^ School of Public Health, Chinese Academy of Medical Sciences and Peking Union Medical College, Beijing, PR China; ^3^ Department of Gynecological Oncology, Chongqing Cancer Institute & Hospital & Cancer Center, Chongqing, PR China; ^4^ Department of Pathology, National Cancer Center, Cancer Hospital, Chinese Academy of Medical Sciences and Peking Union Medical College, Beijing, PR China; ^5^ Department of Gynecology and Obstetrics, The West China Second University Hospital, Sichuan University, Chengdu, PR China

**Keywords:** HPV, p16/Ki-67 dual staining, p16/mcm2, cervical cancer screening, cytology

## Abstract

Most human papillomavirus (HPV) infections are transient and additional triage approaches should be built after HPV-based primary cervical cancer screening. We evaluated the accuracy of p16/Ki-67 and p16/mcm2 dual staining as biomarkers for triaging HPV positive women in China. 4070 participants aged 35 to 64 years attending ongoing cervical cancer screening were enrolled in 2015-2016. Cervical exfoliated cells were collected for HPV DNA analysis and the residual positive specimens were tested for liquid-based cytology and biomarkers. Women infected with HPV 16/18 type or other 12 high-risk HPV types with abnormal cytology results received colposcopy. We found the positive rates of both biomarkers increased significantly with histology severity. p16/Ki-67 positivity in HPV16/18 group, other 12 high-risk HPV group and HPV negative group was 50.0%, 33.7% and 8.9%, respectively. The corresponding p16/mcm2 positivity was 70.0%, 56.3% and 6.7%, respectively. The sensitivity and specificity of p16/Ki-67 for CIN2+ in all HPV-positive women were 91.7% and 63.5%, with a referral rate of 36.2%, while p16/mcm2 were 87.5% and 42.1%, with a referral rate of 58.4%, respectively. The sensitivity of p16/Ki-67 increased to 95.8% for CIN2+ and 100% for CIN3+ when combined with high-grade cytology, without decrease in specificity. Our studies suggest that p16/Ki-67 is an efficient triaging biomarker for HPV-positive women and could reduce colposcopy workload. p16/mcm2 is more sensitive compared with cytology for identifying cervical lesions.

## INTRODUCTION

As the world's most populous developing country, China still faces serious disease burden of cervical cancer, especially in medical resource limited rural areas where reside more than 70% population of the whole country [1]. Despite the current human papillomavirus (HPV) vaccines (Gardasil, Merck; Cervarix, GlaxoSmithKline) targeting HPV 16 and 18 types which cause most cervical cancers [2], they can't protect against established infections nor other HPV types. Screening remains the central method for decades to prevent cervical cancer. High-risk HPV (HR-HPV) testing provides more sensitivity but less specificity compared with cytology for detecting cervical precancer [3–5]. Most HPV infections are transient and usually clear spontaneously in several years and a single HPV DNA test can't discriminate transforming infections from transient ones. Obviously, it's not feasible to send all HPV-positive women to colposcopy which should be prioritized for those with the highest risk for harboring cancer. New programs must be built after primary HPV testing to decide which portion of the HPV-positive women should receive colposcopy immediately and meanwhile delay colposcopy to reduce transient HPV infections for those with relatively low risk of progressing to precancer [6].

Several studies have demonstrated that cytology provides more sensitivity for detection of precancer when informed of HPV status compared to a single cytology test [7, 8]. Therefore, a widely recommended suggestion is to move cytology into the role of triage in HPV primary screening [9]. However, in spacious rural regions around China, lack of essential health infrastructure makes it fairly difficult to store cytologic specimens and train experienced cytopathologists to interpret these cytology slides [1]. In face of various limitations to implement Liquid-based cytology (LBC) programs, finding more appropriate and objective triage approaches for the management of HPV-positive women becomes a critical issue.

p16^INK4a^ (abbreviated as p16) is a cyclin-dependent kinase (CDK) inhibitor and can be strongly over expressed in transforming HPV infections. As a negative regulator of cell proliferation, p16 protein can down-regulate the activity of CDK4 and CDK6 when the retinoblastoma protein has been inactivated [10, 11]. Previous studies have shown that p16 could be served as a surrogate marker for precancerous cervical lesions [12, 13]. As a nuclear antigen and cellular proliferation biomarker, Ki-67 can express exclusively in nucleus of proliferating cells during all cell-cycle stages except G_0_ [14]. Minichromosome maintenance protein 2 (mcm2) participates in DNA replication in all eukaryotic cells. It can promote cell proliferation by loading the complex onto DNA and unwinding the DNA helicase to permit DNA synthesis [15, 16]. Recently, an immunocytochemical assay called ProEx^TM^C composed of 2 antibodies against mcm2 and topoisomerase IIα (TOP2A) has been selected as promising diagnostic adjunct for cervical squamous intraepithelial lesions [17, 18].

So far, no studies have combined a cocktail reagent simultaneous containing p16 and mcm2 antibodies just as CINtec PLUS (Roche, Tucson, AZ, USA) targeted for p16/Ki-67 co-expression. As cell-cycle related acceleration proteins, there may be potential overlaps in function between Ki-67 and mcm2. With this assumption, we performed this study to evaluate the clinical performance of p16/Ki-67 and a novel p16/mcm2 dual-staining cytology approach with the focus on triaging HPV-positive women.

## RESULTS

Among 4,070 participating women with complete HPV data, 357 (8.8%) were positive for HPV DNA. Liquid-based cytology was obtained for all HPV-positive women and 252 (70.6%) of 357 were normal, 46 (12.9%) were atypical squamous cells of undetermined significance (ASC-US), 23 (6.4%) were low-grade squamous intraepithelial lesions (LSILs), 4 (1.1%) were atypical squamous cells that cannot exclude high-grade lesion (ASC-H), 4 (1.1%) were high-grade squamous intraepithelial lesions (HSILs), and 28 (7.8%) were unsatisfactory (including 4 HPV 16/18 types and 24 other HR-HPV types) due to insufficient sample material which we decided to exclude. A total of 329 eligible dual-staining tested, 119 (36.2%) were p16/Ki-67 positive, 192 (58.4%) were p16/mcm2 positive.

**Figure 1:**
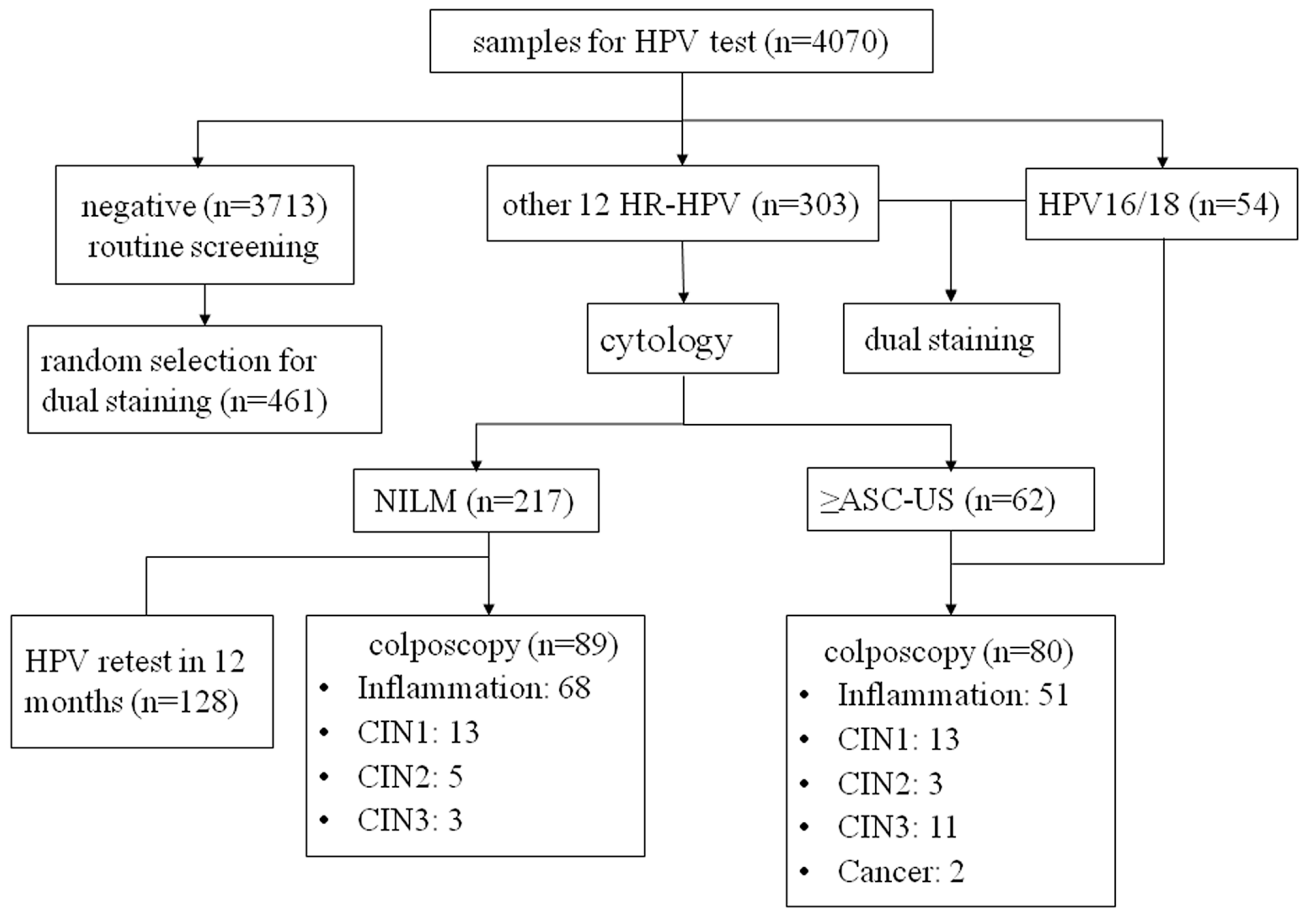
Flow diagram showing procedures involved in every step of the study

Women infected with HPV 16/18 type (n=50) or other carcinogenic types with abnormal cytology results (n=62) were referred to immediate colposcopy while 32 women refused. In the 80 colposcopies performed, 3 cases of CIN2, 9 cases of CIN3 and 2 squamous carcinoma were found. There were also 5 cases of CIN2, 3 cases of CIN3 in HPV-positive, cytology-negative women who received colposcopy. A total of 169 women had complete histology results. We compared the baseline characteristics of women for compliance and non-compliance to colposcopy (Table 1) and found that except for ages, there were no significant differences among HPV infection status, p16/Ki-67 and p16/mcm2 positive rates. In addition, we conducted a random selection of 12% (n=461) among HPV negative women to perform liquid-based cytology as well as p16/Ki-67 and p16/mcm2 dual-staining. A total of 790 women were included in the final analysis. Figure 1 shows the procedure of our study.

**Table 1:** Baseline characteristics of women in this study by colposcopy compliance

Characteristics at baseline	Colposcopy accepted (n=169)	Colposcopy refused (n=32)	*p* Value
**Age**			
Average age	45.2±5.6	49.3±7.2	<0.001
≤45	89 (52.7%)	10 (31.2%)	0.026
≥46	80 (47.3%)	22 (68.8%)	
** HPV status**			
HPV16/18 type	38 (22.5%)	12 (37.5%)	0.072
Other HR-HPV types	131 (77.5%)	20 (62.5%)	
**p16/Ki-67 dual staining**			
Positive	75 (44.4%)	14 (43.7%)	0.948
Negative	94 (55.6%)	18 (56.3%)	
**p16/mcm2 dual staining**			
Positive	105 (62.1%)	19 (59.4%)	0.769
Negative	64 (37.9%)	13 (40.6%)	

As shown in Table 2, the overall positivity of p16/Ki-67 (20.3%) and p16/mcm2 (28.2%) dual staining were higher than LBC (10.2%) and HPV16/18 genotyping (6.3%) in the whole population (*p*<0.05). Positivity rates of both p16/Ki-67 and p16/mcm2 dual staining increased significantly with histology severity (*p*_trend_<0.001). There were 119 (70.4%) women with inflammation, 26 (15.4%) with CIN1, 8 (4.7%) with CIN2, 14 (8.3%) with CIN3 and 2 (1.2%) with squamous cervical cancer. The corresponding p16/Ki-67 positive rates were 34.4%, 46.2%, 87.5%, 92.9%, and 100%, respectively, while p16/mcm2 were 56.3%, 65.4%, 75.0%, 92.9%, and 100%, respectively.

**Table 2:** p16/Ki-67, p16/mcm2, LBC and HPV16/18 positivity in cytology and histology categories

Category	N	p16/Ki-67 N (%)	p16/mcm2 N (%)	LBC(≥ ASC-US) N (%)	HPV16/18 N (%)
**Cytology**					
NILM	709	113 (15.9)	170 (24.0)	-	35 (4.9)
ASC-US	48	28 (58.3)	32 (66.7)	-	5 (10.4)
ASC-H	4	2 (50.0)	3 (75.0)	-	1 (25.0)
LSIL	25	14 (56.0)	14 (56.0)	-	6 (24.0)
HSIL	4	3 (75.0)	4 (100)	-	3 (75.0)
**Histology**					
Inflammation	119	41 (34.4)	67 (56.3)	33 (27.7)	23 (19.3)
CIN1	26	12 (46.2)	17 (65.4)	8 (30.8)	8 (30.8)
CIN2	8	7 (87.5)	6 (75.0)	3 (37.5)	1 (12.5)
CIN3	14	13 (92.9)	13 (92.9)	9 (64.3)	5 (35.7)
SCC	2	2 (100)	2 (100)	1 (50)	1 (50)
Follow-up	621	85 (13.7)	118 (19.0)	27 (4.3)	12 (1.9)
Total	790	160 (20.3)	223 (28.2)	81 (10.2)	50 (6.3)

NILM, no cervical intraepithelial neoplasia or malignancy; ASC-US, atypical squamous cells- undetermined significance; ASC-H, atypical squamous cells cannot exclude high-grade squamous intraepithelial lesion; LSIL, low-grade squamous intraepithelial lesion; HSIL, high-grade squamous intraepithelial lesion; SCC, squamous cervical cancer; LBC, liquid-based cytology; CIN, cervical intraepithelial neoplasia.

All participants were divided into three groups based on HPV infection status to compare the positive rates of both biomarkers in different HPV types. Table 3 showed that p16/Ki-67 positive rates in both HPV 16/18 group (50.0%) and other 12 HR-HPV group (33.3%) were significantly higher compared with HPV negative group (8.9%), similar results were also observed in p16/mcm2 dual staining. Besides, p16/Ki-67 positivity was significantly higher in HPV 16/18 type than other HR-HPV types (*p*=0.024), but no differences were found for p16/mcm2 expression rate in these two groups (*p*=0.063).

**Table 3:** Positive rates of p16/Ki-67, p16/mcm2 dual staining in different HPV infection status

HPV status	p16/Ki-67			p16/mcm2		
	N (%)	χ^2^	*P*	N (%)	χ^2^	*P*
HR-HPV negative (n=461)	41 (8.9)	ref	-	31 (6.7)	ref	-
Other 12 HR-HPV positive (n=279)	94 (33.7)	71.7	<0.01	157 (56.3)	225.2	<0.01
HPV16/18 positive (n=50)	25 (50.0)	67.8	<0.01	35 (70.0)	160.6	<0.01

Other 12 HR-HPV positive: positive for any of the 12 HPV types (HPV 31, 33, 35, 39, 45, 51, 52, 56, 58, 59, 66 and 68), and negative for HPV16/18.

Sensitivities, specificities, PPVs, NPVs and colposcopy referral rates for all HPV-positive women to detect CIN2+ and CIN3+ were evaluated (Table 4). The sensitivity of p16/Ki-67 dual staining to detect CIN2+ and CIN3+ were 91.7% and 93.8%, while p16/mcm2 were 87.5% and 93.8%, respectively. Sensitivities of both biomarkers for detection of CIN2+ and CIN3+ were significantly higher than LBC and HPV16/18 genotyping (all *p*<0.05). The specificity of p16/Ki-67 for both endpoints were similar to LBC (63.5% vs 71.7% for CIN2+, 60.8% vs 71.2% for CIN3+, all *p*>0.05) and lower than HPV16/18 genotyping (63.5% vs 78.6% for CIN2+, 60.8% vs 79.1% for CIN3+, all *p*<0.05). Compared with LBC and HPV16/18 genotyping, p16/mcm2 had a lower specificity for both CIN2+ and CIN3+. Notably, no significant differences of sensitivity were found between p16/Ki-67 and p16/mcm2 dual staining, but p16/Ki-67 had a higher specificity for both endpoints (63.5% vs 42.1% for CIN2+, 60.8% vs 41.2% for CIN3+, all *p*<0.05). In addition, the positivity of p16/Ki-67 in HR-HPV positive women was 36.2%, indicating that more than 60% of women needn't receive colposcopy if p16/Ki-67 dual staining was used as a triage test and meanwhile women who performed p16/mcm2 test would also lead a referral rate decline by more than 40%.

**Table 4:** Clinical performance of p16/Ki-67, p16/mcm2, LBC, HPV16/18 genotyping and other 12 HR-HPV for detection of CIN2+ and CIN3+ in women with positive HPV results

Triaging methods	Sensitivity % (95% CI)	Specificity % (95% CI)	PPV % (95% CI)	NPV % (95% CI)	Referral rate %(95% CI)
CIN2+ (n=24)					
p16/Ki-67	91.7 (74.2-97.7)	63.5 (55.4-70.9)	29.3 (20.2-40.4)	97.9 (92.6-99.4)	36.2 (31.1-41.5)
p16/mcm2	87.5 (69.0-95.7)	42.1 (34.3-50.2)	20.0 (13.5-28.7)	95.3 (87.1-98.4)	58.4 (53.0-63.6)
LBC	54.2 (35.1-72.1)	71.7 (63.9-78.4)	24.1 (14.6-37.0)	90.4 (83.7-94.6)	23.4 (19.1-28.2)
HPV16/18 genotyping	29.2 (14.9-49.2)	78.6 (71.3-84.5)	18.4 (9.2-33.4)	87.0 (80.2-91.7)	15.2 (11.6-19.4)
Other 12 HR-HPV	70.8 (50.8-85.1)	21.4 (15.5-28.8)	13.0 (8.3-19.8)	81.6 (66.6-90.8)	35.3 (32.1-38.7)
CIN3+(n=16)					
p16/Ki-67	93.8 (71.7-98.9)	60.8 (52.9-68.2)	20.0 (12.5-30.4)	98.9 (94.2-99.8)	36.2 (31.1-41.5)
p16/mcm2	93.8 (71.7-98.9)	41.2 (33.7-49.1)	14.3 (8.9-22.2)	98.4 (91.7-99.7)	58.4 (53.0-63.6)
LBC	62.5 (38.7-81.5)	71.2 (63.6-77.8)	18.5 (10.4-30.8)	94.8 (89.1-97.6)	23.4 (19.1-28.2)
HPV16/18 genotyping	37.5 (18.5-61.4)	79.1 (72.0-84.8)	15.8 (7.4-30.4)	92.4 (86.5-95.8)	15.2 (11.6-19.4)
Other 12 HR-HPV	62.5 (38.6-81.5)	20.9 (15.2-28.0)	7.6 (4.2-13.5)	84.2 (69.6-92.6)	35.3 (32.1-38.7)

CIN2+, cervical intraepithelial neoplasia grade 2 or worse; CIN3+, cervical intraepithelial neoplasia grade 3 or worse; CI, confidence interval; PPV, positive predictive value; NPV, negative predictive value.

We also evaluated the clinical performance of biomarkers for triaging women with HPV-positive, cytology-negative results. Positivity rates of p16/Ki-67 and p16/mcm2 among 252 HPV-positive, cytology-negative women were 29.0% and 55.6%, respectively. There were 11 CIN2+ and 6 CIN3+ cases diagnosed during preliminary follow-up (Table 5). The sensitivity of p16/Ki-67 and p16/mcm2 for CIN2+ were 90.9% and 81.8%, with a specificity of 71.7% and 46.2%, respectively. Notably, for a CIN3+ threshold, the sensitivity of both biomarkers reached to 100%, with a specificity of 69.4% and 46.0%, respectively.

**Table 5:** Performance of p16/Ki-67 and p16/mcm2 for triaging HPV-positive, cytology-negative women

Endpoint	Test	Sensitivity, % (95% CI)	Specificity, % (95% CI)	PPV, % (95% CI)	NPV, % (95% CI)
CIN2+ (n=11)	p16/Ki-67	90.9 (62.3-98.4)	71.7 (62.5-79.4)	25.0 (14.2-40.2)	98.7 (93.0-99.8)
	p16/mcm2	81.8 (52.3-94.9)	46.2 (37.0-55.7)	13.6 (7.3-23.9)	96.1 (86.8-98.9)
CIN3+ (n=6)	p16/Ki-67	100 (61.0-100)	69.4 (60.3-77.2)	15.0 (7.1-29.1)	100 (95.3-100)
	p16/mcm2	100 (61.0-100)	46.0 (37.0-55.2)	9.1 (4.2-18.5)	100 (93.0-100)

CIN2+, cervical intraepithelial neoplasia grade 2 or worse; CIN3+, cervical intraepithelial neoplasia grade 3 or worse; CI, confidence interval; PPV, positive predictive value; NPV, negative predictive value.

Combined strategies of cytology at a HSIL threshold, biomarkers and HPV16/18 genotyping in all HPV-positive women were shown in Table 6. We found that adding HSIL to p16/Ki-67 dual staining and HPV16/18 genotyping could increase sensitivity for both endpoints, without loss of specificity. For instance, the sensitivity of combined p16/Ki-67 and HSIL were 95.8 for CIN2+ and 100% for CIN3+,while the specificity were 63.5% and 60.8, respectively. However, no differences were found when cytology was combined with p16/mcm2 for CIN2+, but the sensitivity has increased to 100% for CIN3+, without decrease in specificity. Combined strategies of HSIL and HPV16/18 genotyping reached a sensitivity of 33.3% for CIN2+ and 43.8% for CIN3+, respectively.

**Table 6:** Combined strategies of biomarkers, cytology and HPV16/18 genotyping for triaging HPV-positive women

Triaging methods	Sensitivity % (95% CI)	Specificity % (95% CI)	PPV % (95% CI)	NPV % (95% CI)
CIN2+ (n=24)				
p16/Ki-67 and HSIL	95.8 (79.8-99.3)	63.5 (55.4-70.9)	30.3 (21.1-41.3)	98.9 (94.2-99.8)
p16/mcm2 and HSIL	87.5 (69.0-95.7)	42.1 (34.3-50.2)	20.0 (13.5-28.7)	95.3 (87.1-98.4)
HPV16/18 and HSIL	33.3 (18.0-53.3)	78.6 (71.3-84.5)	20.5 (10.8-35.5)	87.7 (80.9-92.3)
CIN3+ (n=16)				
p16/Ki-67 and HSIL	100 (80.6-100)	60.8 (52.9-68.2)	21.1 (13.4-31.5)	100 (96.0-100)
p16/mcm2 and HSIL	100 (80.6-100)	41.2 (33.7-49.1)	15.1 (9.5-23.1)	100 (94.3-100)
HPV16/18 and HSIL	43.8 (23.1-66.8)	79.1 (72.0-84.8)	18.0 (9.0-32.7)	93.1 (87.4-96.3)

HSIL, high-grade squamous intraepithelial lesion; CIN, cervical intraepithelial neoplasia; CI, confidence interval; PPV, positive predictive value; NPV, negative predictive value.

## DISCUSSION

Recent guidelines in the United States [19] and the new European GGLL [20] have recommended HPV testing with cytology triage as the primary cervical cancer screening strategy, instead of co-testing. Typing with HPV 16 or 18 of women even with negative cytology results have high enough risk for direct referral to colposcopy while other HR-HPV types with normal cytology result are followed up after 12 months [21]. This algorithm has eliminated the group of HPV-negative ASC-US results which in fact constitute a large proportion of abnormal cytology in primary screening. However, restricting cytology to HPV-positive women has actually little or even no increased risk of precancer, for a negative HPV result can almost exclude women with precancer or cancer [22].

To the best of our knowledge, this is the first population-based study in China comparing triage strategies with cocktail antibodies of p16/Ki-67 and p16/mcm2 in HPV-based primary screening. To realize a good quality control, those classified as abnormal cytology or worse (ASC-US+) and a 10% random selection of normal slides were reviewed by an expert pathologist. Meanwhile, four-quadrant cervical biopsies and endocervical curettage (ECC) were performed to maximize the diagnosis of disease in case of no visible lesions under colposcopy. Besides, all dual staining tests in our study were performed in the central laboratory according to the same criteria by using the same sample.

In our study, we observed a significantly increased positive rates of both p16/Ki-67 and p16/mcm2 with the increasing grade of histology severity, which were similar to previous studies [23, 24]. Theoretically, concomitant expression of p16 and Ki-67 is mutually exclusive within one cell, which may only occur after impairment of the cell-cycle control mechanism and thus simultaneous p16 and Ki-67 expression in the same cell can be treated as an indicator of cell-cycle deregulation [25, 26]. Previous studies have revealed the overexpression of mcm2 in cervical cancer by DNA microarray and transcriptional profiling [27], showing that mcm2 can be detected in various dysplastic and malignant processes, including cervical neoplasia related to high-risk HPV [28]. Our study demonstrated a strong association between p16/Ki-67, p16/mcm2 expression and the status of HPV infection. The positivity of both biomarkers were significantly higher in HPV positive group compared with negatives, especially in HPV16/18 positive group, which were consistent with previous study [29]. One possible explanation may be that HPV 16 and 18 types have a closer association with invasive cervical cancer and represent an elevated risk of high-grade CIN, with higher potential to secrete HPV16/18 oncoproteins [30, 31].

We found that p16/Ki67 was positive in 36.2% of all HPV-positive women, with a sensitivity of 91.7% for CIN2+ and 93.8% for CIN3+, indicating that it could detect most cervical precancer and meanwhile reduced unnecessary colposcopy referrals. The sensitivity of p16/Ki-67 in our study was significantly higher than LBC and HPV16/18 genotyping, in accordance with previous studies [32, 33]. It represented a similar specificity for CIN2+ compared to LBC, which was inconsistent with other research [29]. It may be due to different study design and population. Furthermore, the PPV of p16/Ki-67 was similar to LBC and 2-fold higher than other12 HR-HPV types. It was even higher than HPV16/18 genotyping, although the latter had better specificities for both endpoints, indicating the feasibility of treating p16/Ki-67 as a triage test, or at least triage women with other 12 HR-HPV types. Since in Chinese rural areas, where medical resource is very limited, the broad use of LBC is unfeasible. Notably, our study explored an innovational cocktail antibodies: p16/mcm2 dual staining, which has not been reported before. In our study, the sensitivity of p16/mcm2 was significantly higher than LBC and HPV16/18 genotyping and meanwhile very close to p16/Ki-67, especially for CIN3+ endpoint. Despite the low specificity compared with cytology, p16/mcm2 could cut off the referral rate by more than 40%, instead of referring all HR-HPV women to colposcopy.

Interestingly, we evaluated the performance of both biomarkers in women with HPV-positive, cytology-negative results and found that p16/Ki-67 dual staining represented better sensitivity and comparable specificity than former studies [34, 35], especially for CIN3+ endpoint with a sensitivity reaching to 100%. It should be mentioned that the novel p16/mcm2 dual staining in our study showed similar sensitivity for CIN3+ compared with p16/ProE_X_ C from previous studies [24]. It means that both p16/Ki-67 and p16/mcm2 are able to identify most CIN2+ cases missed by cytology test, compensating the sensitivity deficiency of Pap cytology.

Our study demonstrated that combined strategies of cytology and biomarkers have an increased sensitivity and moderate specificity only when cytology adopted a high-grade threshold. Meaning that the biomarkers could increase specificity by triaging women with ASC-US and LSIL results and increase sensitivity by triaging women with NILM results. Our results showed very low sensitivity of HPV16/18 genotyping, similar to previous study [30], and thus referring only HPV 16 and 18 to colposcopy hasn't been accepted by any protocols. However, adding HSIL as a referral criterion to HPV 16/18 could increase the sensitivity without any decrease in specificity, instead of a ASC-US threshold, which slightly increased the sensitivity with a substantial loss in specificity.

The main limitation of our study is that we can't have a colposcopy assessment for all HPV-positive women. Since the study was nested in the pragmatic process of an organized national program. According to the study design, only cytology positive women received colposcopy and part of women with NILM results were followed up after 1 year. This differential ascertainment led to a bias in favor of cytology sensitivity. Our study focused on the cross-sectional performance of biomarkers even though cervical precancer includes a certain regressive lesions during natural history, not distinguishable from progressive ones. But the relevance of regression has limited because we recruited only 35 years or older women. Furthermore, we evaluated the prognostic value of biomarkers only in HPV-positive, cytology negative women, without containing the longitudinal data of CIN in women with normal triage results.

In summary, p16/Ki-67 had a higher sensitivity and comparable specificity compared with cytology, and could increase sensitivity for both endpoints when combined with high-grade cytology. p16/mcm2 was more sensitive than cytology for identifying cervical lesions. Furthermore, both biomarkers could recognize cervical lesions simply based on dual-staining within one cell which potentially eliminate the morphological criterion, suitable for Chinese rural areas where experienced cytopathologists aren't available. It seems p16/Ki-67 is more promising to reduce colposcopy workload and allows longer intervals in HPV positive, biomarker negative women.

## MATERIALS AND METHODS

### Study population

From April 2015 to May 2016, 4,070 eligible women in the districts of Wanzhou (Chongqing, China) and Shuangliu (Sichuan, China) with a median age of 47.5 years agreed to participate in a HPV-based cervical cancer screening. The screening strategy at each participating center was implemented in accordance with the approved guidelines. All participants have signed up informed consent forms and this study was approved by the institutional review boards of the Cancer Institute/Hospital, Chinese Academy of Medical Sciences (CICAMS) in Beijing and the local ethical committee (The Second Affiliated Hospital, Sichuan University; Chongqing Cancer Hospital). All participants should meet the following criteria: aged between 35-64 years; without history of previous cervical diseases and had an intact cervix; were not pregnant; understood the study procedure and were able to provide informed consent.

### Study procedures

This was a population-based study within the setting of the national cervical cancer program searching for suitable screening technologies for Chinese rural areas. After completing a questionnaire containing various risk factors by well-trained health workers, one cervical cytology specimen was collected for each participant with a cytobrush by physicians and then transferred to PreservCyt solution (Hologic Inc., Bedford, MA), stored at 4°C, and transported to central lab for primary HPV DNA testing. Women negative for HPV will return to routine screening with a 3-year interval. For each of the HPV positive women, a thin-layer slide for LBC was prepared and read. Women who tested positive for HPV16/18 type or other 12 HR-HPV types (31, 33, 35, 39, 45, 51, 52, 56, 58, 59, 66, 68) with ASC-US or worse were referred to colposcopy and biopsy, while those with normal cytology were followed-up and re-tested in 12 months (Figure 1). For all HPV positive women and a 12% random selection of HPV negative at baseline, p16/Ki-67 (MXB, Fuzhou, China) and p16/mcm2 (MXB, Fuzhou, China) immunocytochemical dual staining were performed on residual ThinPrep material, whereas the management for HPV-positive women was not based on the dual stain results. All HPV DNA tests and cytology slides were conducted on the same sample.

### HPV DNA testing

A 1ml aliquot removed from ThinPrep cytology specimens was detected for high-risk HPV using HPV Genotyping Real time PCR Kit (Liferiver, Shanghai, China) which detects viral DNA by nucleic acid hybridization with a pooled probe set for 14 HR-HPV types, including HPV16 type and HPV18 type as well as other 12 high-risk types (31, 33, 35, 39, 45, 51, 52, 56, 58, 59, 66 and 68). All PCR reactions were performed in a 40-ul volume using the ABI PRISM 7000 (Applied Bio-systems, US). Each individual reaction contained 36ul mixture of 2 × TaqMan universal PCR master mix with uracil-N-glycosylase (Applied Bio-systems, US) and two fluorescent probes. The amplification profile was initiated by a 2-minute incubation at 94°C, followed by a two-step amplification of 10 seconds at 93°C and 31 seconds at 62°C for 40 cycles. Data were collected at the end of the amplification process and all experiments were performed in triplicate including positive and non-template controls according to the instructions of the manufacturer.

### Liquid-based cytology

Thin-layer cytology slides were prepared with the ThinPrep 2000 System (Hologic, Boxborough, MA). The preparation process adopted the common gynecology sample procedure: the cell suspension was firstly homogenized after the PreservCyt container placed in the instrument and then cells were collected through a filter membrane to a glass slide. Cells were fixed with 95% ethanol and stained using the Papanicolaou method. Cytology slides were interpreted by two experienced cytotechnologists and confirmed by a third pathologist using the classification of the 2001 Bethesda reporting system. Positive cytology results were defined as atypical squamous cells of undetermined significance or worse, which led to referral to colposcopy.

### Immunocytochemistry dual staining

A second and third monolayer slides were prepared from the residual PreservCyt material by ThinPrep 2000 system in parallel to perform p16/Ki-67 and p16/mcm2 immunocytochemistry dual staining. Immunostaining for p16/Ki-67 was performed using the p16/Ki-67 Detection KIT (Immunocytochemistry, MXB, Fuzhou, China), which contains an antibody cocktail comprising of a mouse monoclonal antibody (clone MX007) directed at p16 protein and a rabbit monoclonal antibody (clone MIB-1) against Ki-67 protein. Secondary antibodies included goat antimouse antigen-binding antibody fragments for detection of p16 with horseradish peroxidase (HRP) and goat antirabbit antibody fragments for detection of Ki-67 with a polymer reagent conjugated to alkaline phosphatase (AP). HRP-mediated conversion of 3,3’-diaminobenzidine (DAB) chromogen, and AP-mediated conversion of Fast Red chromogen emerged the red and yellow-brown staining of p16 and Ki-67, respectively. Finally, we used hematoxylin to counterstain cytology slide with a 3-step mounting procedure including 85%, 95% and pure ethanol medium, each for 1 minute. p16/mcm2 dual staining was performed using antibody cocktail composed of p16 antibody (clone MX007) and rabbit anti-human mcm2 monoclonal antibody (clone SP85) according to the manufacture's instructions. Other procedures were similar to p16/Ki-67 dual staining as the above described. Slides with at least one cervical epithelial cell that simultaneously showed red cytoplasmic immunostaining (p16) and brownish nuclear immunostaining (Ki-67) or moderate to intense yellow-brown nuclear staining (mcm2) were defined as positive results irrespective of the morphological features, while those without any double-stained cells were deemed as negative results. All slides were coverslipped for viewing by a trained cytotechnologist under a light microscope blindly to other testing results.

### Colposcopy and histopathology

Colposcopy processing was conducted in the local reference clinic of the screening program within one month. Colposcopy-directed biopsy specimens were always collected in case of visible cervical lesions and endocervical curettage was performed if the squamocolumnar junction was invisible or suspicious of endocervical lesion. H&E-stained slides were evaluated using the CIN systems according to the current World Health Organization classification. Histopathological diagnosis was firstly made by local pathologists and then all the CIN and a 10% random sample of negative slides were selected to be reviewed by a panel of senior pathologists from corresponding superior hospitals (Chongqing Cancer Hospital and the Second Affiliated Hospital of Sichuan University). Women were regarded as negative histology results if a biopsy need not be taken or the histology diagnosis was normal or CIN1, while those CIN2, CIN3, squamous cell carcinoma (SCC) and adenocarcinoma (ADC) results were defined as CIN2+ cases. The final diagnosis was based on the reading of the panel review regardless of directed biopsies or endocervical curettage.

### Statistical analysis

The study targeted the recruited women with complete screening, triage and diagnostic confirmation results. Diagnostic accuracy of p16/Ki-67 and p16/mcm2 dual staining for CIN2+ and CIN3+ were evaluated by sensitivity, specificity, positive predictive value (PPV) and negative predictive value (NPV), with 95% confidence intervals (CIs). Chi-square of trend for proportion was calculated to test linear associations between triaging methods and increasing severity of histological diagnoses. Associations between biomarkers and HPV status were compared using Chi-square test. McNemar tests were used to compare paired matching data. All data was conducted using SPSS version 19.0 (IBM, Armonk, NY, USA) with statistically significant level of 0.05 (two-sided).
